# Using GPS telemetry to validate least-cost modeling of gray squirrel (*Sciurus carolinensis)* movement within a fragmented landscape

**DOI:** 10.1002/ece3.638

**Published:** 2013-06-12

**Authors:** Claire D Stevenson, Mark Ferryman, Owen T Nevin, Andrew D Ramsey, Sallie Bailey, Kevin Watts

**Affiliations:** 1 University of CumbriaNewton Rigg, Penrith, Cumbria, CA11 0AH, U. K; 2 Forest ResearchFarnham, U. K; 3 Central Queensland UniversityGladstone, Australia; 4 Forestry Commission GBEdinburgh, U. K

**Keywords:** Conservation, corridors, GIS, management

## Abstract

In Britain, the population of native red squirrels *Sciurus vulgaris* has suffered population declines and local extinctions. Interspecific resource competition and disease spread by the invasive gray squirrel *Sciurus carolinensis* are the main factors behind the decline. Gray squirrels have adapted to the British landscape so efficiently that they are widely distributed. Knowledge on how gray squirrels are using the landscape matrix and being able to predict their movements will aid management. This study is the first to use global positioning system (GPS) collars on wild gray squirrels to accurately record movements and land cover use within the landscape matrix. This data were used to validate Geographical Information System (GIS) least-cost model predictions of movements and provided much needed information on gray squirrel movement pathways and network use. Buffered least-cost paths and least-cost corridors provide predictions of the most probable movements through the landscape and are seen to perform better than the more expansive least-cost networks which include all possible movements. Applying the knowledge and methodologies gained to current gray squirrel expansion areas, such as Scotland and in Italy, will aid in the prediction of potential movement areas and therefore management of the invasive gray squirrel. The methodologies presented in this study could potentially be used in any landscape and on numerous species.

## Introduction

Since the introduction of the gray squirrel *Sciurus carolinensis* to Britain, the species has adapted to the British landscape colonizing England, Wales, and parts of Scotland and Ireland (Pepper and Patterson 2001). The population has caused negative effects upon forestry, through damage associated with bark-stripping behavior, and native biodiversity (Kenward 1983; Gurnell and Mayle 2003; Mayle et al. 2007). In particular, the gray squirrel expansion has occurred simultaneously with the decline and replacement of native red squirrel *Sciurus vulgaris* populations. Interspecific competition for resources with the gray squirrel, disease, habitat loss, and fragmentation, are all contributing to the massive decline of the red squirrel within the United Kingdom (Gurnell et al. 2004). In particular, gray squirrel presence in mixed and broadleaved woodland is seen to reduce the reproductive rate and juvenile recruitment of red squirrels (Gurnell et al. 2004). Over time, this results in reduced red squirrel population size and the localized extinction of the red squirrels in that area (Gurnell et al. 2004).

It is suggested that gray squirrels have a decreased sensitivity to habitat fragmentation compared to other Sciurid species (Koprowski 2005), and are capable of crossing all, but the most extreme of land cover types (Bryce et al. 2005). Although red and gray squirrels are capable of traversing open ground (Gurnell et al. 2006), evidence suggests that dispersing Sciurid's will commonly use riparian corridors and valley bottoms as dispersal routes with tree cover being the most influencing factor (Middleton 1930; Wauters et al. 1994, 2010; Bakker and Vuren 2004; Gurnell et al. 2006).

Anecdotal evidence, presence data, and radio tracking have shown that linear landscape elements such as hedgerows, tree rows, road verges, fences, and walls are used by red and gray squirrels during interhabitat patch movements (Middleton 1930; Taylor et al. 1971; Fitzgibbon 1993; Wauters et al. 1997; C. D. Stevenson, K. Watts, O. T. Nevin, and A. D. Ramsey, unpubl. data). Gray squirrels may utilize different land cover types and landscape elements to aid movements, nevertheless there are certain landscape types which are more likely to be used. Being able to predict how these are used during gray squirrel movements will aid management efforts.

The landscape between habitat patches, the landscape matrix, is comprised of different land cover types, which may facilitate or impede species movements (Taylor et al. 1993). When faced with habitat fragmentation, the behavioral and physiological interactions with the landscape are important in determining dispersal and movements (Taylor et al. 1993; Ricketts 2001). The perceptual range of a species to detect particular landscape elements mediates decision making whilst dispersing (Zollner and Lima 2005). Where habitat patches are out of a species perceptual range, landscape elements may act as cues directing a species through the heterogeneous landscape (Pe'er and Kramer-Schadt 2008). The permeability of certain landscape features may also be associated with increased security, shelter, and a food resource (Verboom and van Apeldoorn 1990; Zollner 2000; Bakker and Vuren 2004), whereas others may be related to higher predation and mortality risk (Nixon et al. 1980; Tischendorf and Fahrig 2000). Many studies have found that certain permeable landscape features and linear elements may act as stepping stones and corridors for species movement (Nixon et al. 1980; Beier and Noss 1998; Manning et al. 2006; Bailey 2007; Davies and Pullin 2007; Gelling et al. 2007). The effects of habitat fragmentation on species movement are therefore species and landscape specific (Tischendorf and Fahrig 2000).

Many studies have used GIS least-cost modeling to assess the functional connectivity of fragmented habitat patches (Villalba et al. 1998; Ferreras 2001; Adriaensen et al. 2003; Chardon et al. 2003; Coulon et al. 2004; Driezen et al. 2007; Epps et al. 2007; Gonzales and Gergel 2007; Walker et al. 2007; LaRue and Nielsen 2008; Janin et al. 2009; Watts et al. 2010; Sawyer et al. 2011). In particular, Villalba et al. (1998), Verbeylen et al. (2003), and Gonzales and Gergel (2007) all used least-cost modeling to assess connectivity for Sciurid species. Whilst Stevenson et al. (in review) used least-cost modeling to specifically predict gray squirrel movements. During least-cost modeling, land cover types are assigned a resistance or permeability score which is based upon the facilitating or impeding effects upon species movement (Adriaensen et al. 2003). Three types of least-cost models are defined; least-cost networks (LCN), buffered least-cost path (LCP), and least-cost corridor (LCC). LCN identify functional habitat networks which include patches of habitat and a buffer of permeable surrounding landscape which could potentially be utilized for movement based upon defined permeability values and a dispersal distance (Watts et al. 2010). LCP analysis is a common type of least-cost modeling which shows a single least-cost route between a start and end point (Sawyer et al. 2011). Whereas, LCC are formed by combining multiple LCP which are buffered by the landscape resistance values at each side of the LCP. Beier et al. (2008) suggests that LCC are most appropriate for identifying connectivity as they account for alternative movement routes (Beier et al. 2008; LaRue and Nielsen 2008; Sawyer et al. 2011).

This study aims to use a combination of LCN, LCP, and LCC modeling to identify potential gray squirrel movement paths. To assess these alternative least-cost models, and also to add to the current knowledge of gray squirrel landscape movement, this study uses global positioning system (GPS) telemetry to record gray squirrel movements. Gray squirrel movements have been recorded previously by radio telemetry (see Haughland et al. 2008). Although Swihart and Nupp (1998) and Swihart et al. (2007) have investigated matrix usage by gray squirrels, to our knowledge no study has recorded high spatial and temporal resolution gray squirrel movements with GPS devices. The information gained will enable a comparison of alternative LC models and the prediction of gray squirrel movements through a fragmented landscape.

## Material and Methods

### Study site

**Figure 1:**
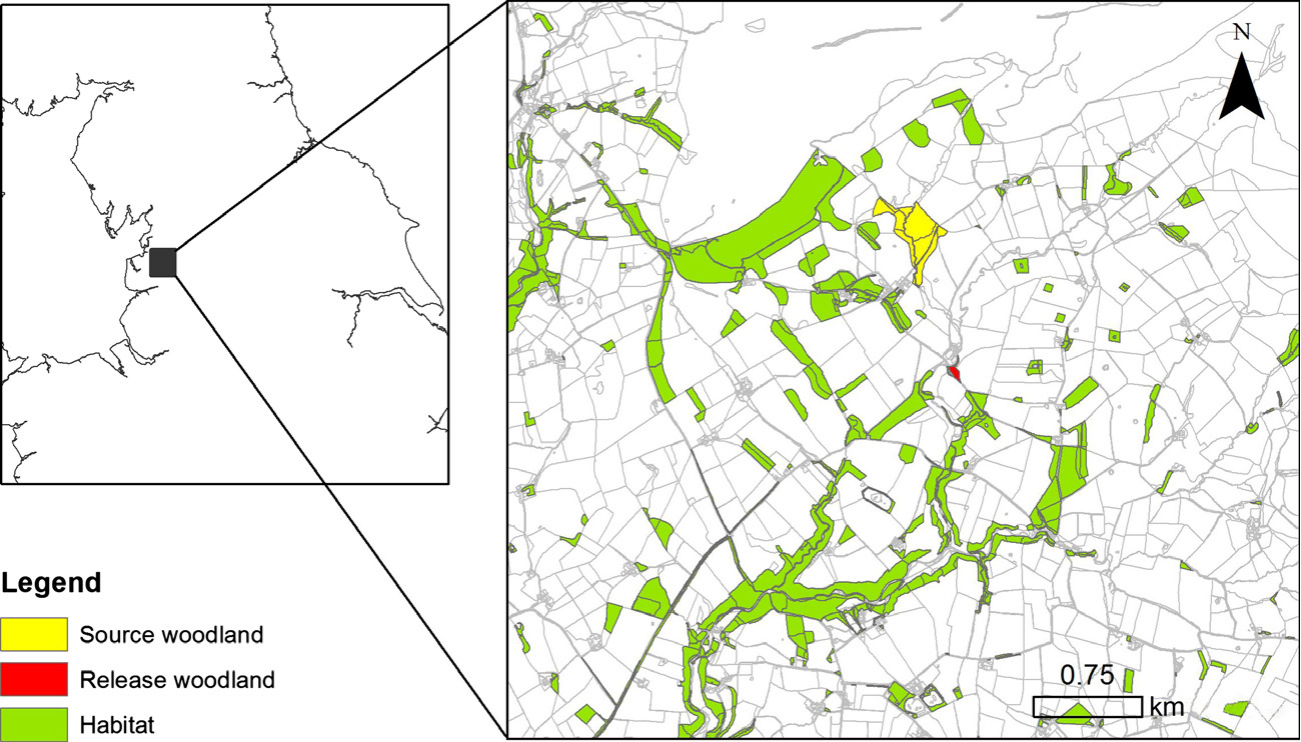
Study site, Lancashire, U.K.

The study site in the County of Lancashire, United Kingdom comprises a variety of different land cover types which could potentially affect gray squirrel movements. Habitat patches are highly fragmented and therefore individuals will need to move into the surrounding landscape matrix to move between habitat patches (Fig. 1). River corridors, road verge, hedgerow, fence row, tree rows, and small habitat patches are all connected to the release woodland giving the gray squirrel land cover and feature options to aid their movements. Due to the vulnerability of red squirrel populations to squirrelpox virus transmission, this study was conducted in an area where no red squirrel populations occur; no red squirrels have been present on the study site for at least 10 years.

### GPS equipment and collaring of gray squirrels

Gray squirrels are a nonnative species listed on Schedule 9 of the Wildlife and Countryside Act 1981 and cannot be released into the wild in the United Kingdom without a license. Prior to the study, a license was obtained from Natural England. A sample wood was chosen as the main source population for gray squirrel individuals (Fig. 1). It consists of 10.88 ha of mixed conifer and broadleaved tree species and is situated within the center of the study site. Squirrels were trapped using 10 Albion squirrel/mink single-capture traps placed in the woodland and prebaited with yellow whole maize for 7 days. Gray squirrel visits to traps were confirmed by examining the part-eaten bait. Trapping, handling, and collaring of squirrels were carried out by workers competent in these skills.

**Figure 2:**
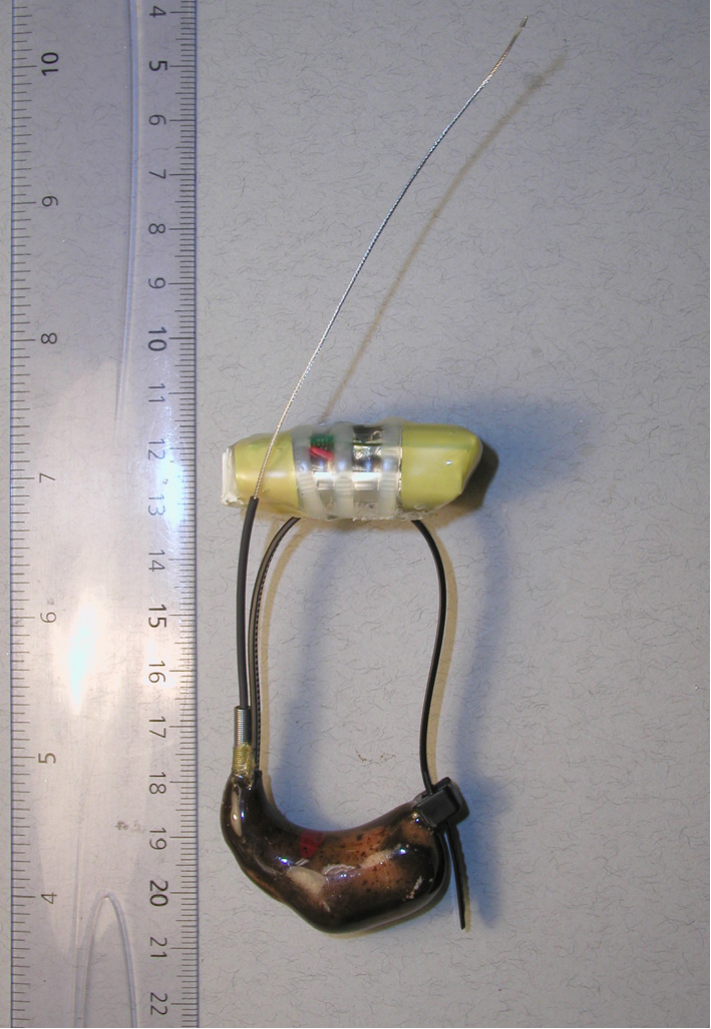
Complete GPS radio collar ready for fitting.

To obtain data on gray squirrel movements in the landscape matrix, locations should ideally be taken at least every few minutes to capture the movement. Squirrel movements were recorded using I-gotU GPS travel trackers (A41JF, Maplin, U.K.) which were modified by the authors to make them durable and waterproof. The devices were removed from their (nonwaterproof) plastic enclosures, completely coated in a UV-resistant waterproof plastic coating and epoxy resin shell. Each modified device weighed about 22 g, near to their original weight. Provision was made to attach the devices to a standard squirrel radio collar (Fig. 2). Initial tests indicated that fully charged devices would operate for approximately 5 days where location data were set to record every 3 min. This was the setting used for the study. As locations are stored in the GPS memory, the recapture of squirrels is necessary to collect the data. To enable this, a very high frequency radio transmitter (LPM-2320, Alana Ecology Ltd, Totnes, U.K.) was also fitted to the collars to enable tracking of individuals. The battery life of the radio transmitter lasts up to 6 months enabling a sufficient time period to recover the GPS devices.

**Figure 3:**
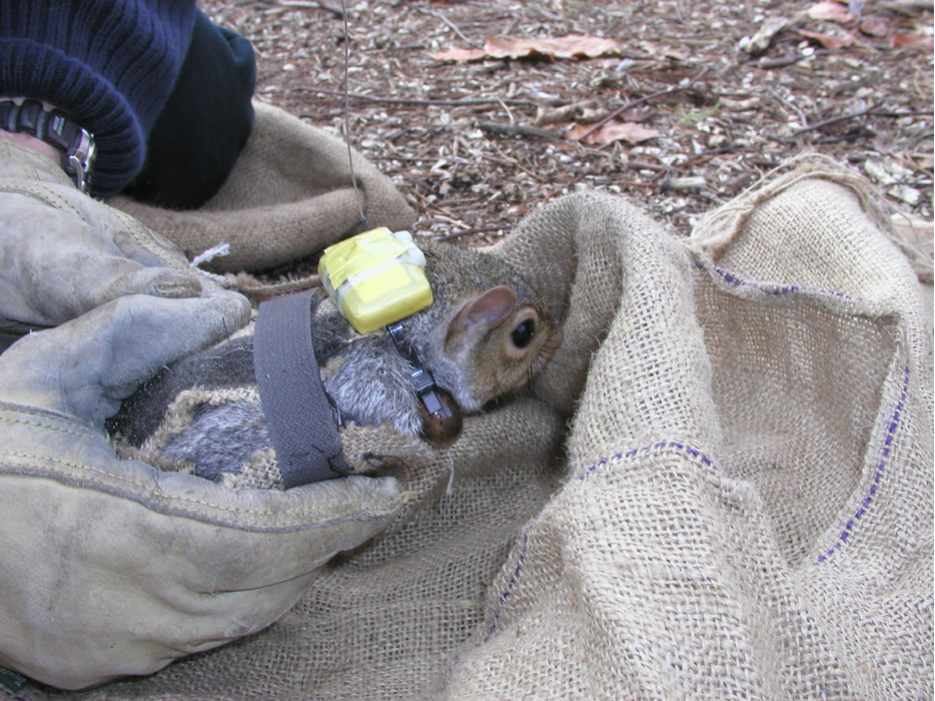
Fitted collar with GPS device at the top and radio transmitter underneath. Note the hessian cone with adjustable neck still to be undone, releasing the squirrel.

To avoid affecting the normal behavior of squirrels, collars should not exceed 4–7% of the bodyweight; the combined radio/GPS collars weighed was 32 g (Fig. 2) as used in Kenward (1982). Squirrels were transferred from a trap into a hessian sack, then into a standard wire mesh handling cone for a health check. Squirrels larger than 460 g were used for the study. For collaring, individuals were transferred to a hessian handling cone enabling the head of the squirrel to be free (Koprowski 2002). This cone design enabled a squirrel to be restrained by one worker while another fitted the collar. Standard wire mesh cones are generally unsuitable due to their constriction and access. The radio transmitter was fitted under the chin of the squirrel and the GPS device on the back of the neck as it needed to face upwards to enable signal transfer (Fig. 3). Before the study, two squirrels were fitted with these collars and released into a squirrel research enclosure (2500 m²) with other uncollared squirrels to observe them in a near-natural environment. The squirrels were observed using CCTV at feeding hoppers and traps for any abnormal behavior and to ensure the GPS unit remained facing upwards.

All squirrels were translocated and released (under Natural England license) in a small mixed broadleaved and coniferous woodland patch of 0.45 ha approximately 500 m away from the source woodland (Fig. 1). This release woodland was surrounded by numerous landscape features and land cover types. Collared individuals were released for 5 days before recapture began. Squirrels were tracked once a day using radio telemetry until captured or the time scale for the study had ended. In this study, each position was taken either by triangulation or by homing in on the radio signal until a visual fix was gained. Traps were placed and set in woodlands where the collared gray squirrel individuals were located. Collars were then removed from the recaptured individuals and the data recovered.

### Defining a movement and assessing land cover use

All data points that were recorded on the GPS devices were entered into ArcGIS (Esri, Redlands, CA). As locations are taken every three minutes, when a squirrel is stationary or remains in a certain area for long periods of time a number of points are recorded at that location. As this study is concentrating on movements within the matrix and not within habitat, consecutive points located between two woodland patches were selected. The ArcGIS Point Density tool calculates the density of points within the landscape and highlights areas that have high-to-low density. This was used to highlight woodlands where the squirrel has spent time in one location compared to points when the squirrel is moving through the landscape. A reviewer suggests that the Brownian Bridge technique may also be used to identify movements. In this study, movement is defined as a number of consecutive points that occur between two woodland patches/highlighted clusters. The first point of the movement is the last point within the cluster and the last movement point is the first point to be recorded within the next cluster. The length of the movement was recorded and compared to the Euclidian distance.

The number of GPS points within each land cover type was calculated to assess usage. The distance from each of the movement points to the nearest habitat patch and the nearest landscape feature was measured within ArcGIS. Habitat edge, river corridor, road, road verge, track, path, and field edge (walls, fences, and hedgerows) are all classed as landscape features. Land cover and feature use were ranked based upon the number of GPS points within each land cover type compared to the expected number of GPS points if distributed proportionally to each land cover type.

### Identifying least-cost networks

A LCN tool (described in Watts et al. 2010) was used to identify the possible movement areas for the gray squirrel. The model identifies habitat networks which indicate areas of the landscape where gray squirrels are likely to move through. Ordnance Survey Master Map (OSMM) data were used as the land cover map with similar land cover types grouped together to provide 21 land cover categories (C. D. Stevenson, K. Watts, O. T. Nevin, and A. D. Ramsey, unpubl. data). Woodlands of all sizes were classed as gray squirrel habitat and 8 km was used as the maximum dispersal distance (as in C. D. Stevenson, K. Watts, O. T. Nevin, and A. D. Ramsey, unpubl. data). Each land cover type was assigned a resistance score or permeability values representing the cost of moving through each land cover type to the study species. These scores were based on the expert-derived scores used in C. D. Stevenson, K. Watts, O. T. Nevin, and A. D. Ramsey (unpubl. data) and were used in the LCN, LCP, and LCC modeling (Table 1). Least-cost models are sensitive to the values entered and changes in scores can affect the resulting network, paths, or corridors. However, the scores used here have previously been evaluated and compared to species distribution data (C. D. Stevenson, K. Watts, O. T. Nevin, and A. D. Ramsey, unpubl. data.)

**Table 1:** Gray squirrel resistance values used in LC models (based on C. D. Stevenson, K. Watts, O. T. Nevin, and A. D. Ramsey, unpubl. data)

Land cover type	Resistance value
Broadleaf	1
Mixed woodland	1
Coniferous	1
Orchard	16
Scrub	16
Coppice	16
Garden	16
I/A/A*	40
Grass	40
Heath	37
Path	27
Railway verge	27
Road verge	27
Marsh	91
Water	130
Urban	72
Railway	55
Road verge	27
Track	27
Building	1000
Rock	1000

* Improved/Arable and Amenity.

### Adding additional features to the land cover map

Due to the fine scale of LCP and LCC, it was necessary to add additional features or small woodland patches to the land cover map as they were not present in the OSMM, but would potentially affect species movements (Villalba et al. 1998; Schadt et al. 2002; Adriaensen et al. 2003). A study site visit indicated that three tree rows had not been included within the original OSMM. As field-edge features were also not represented, these were therefore digitized on to the OSMM. Within this study area, field edges contained fence rows, walls, and hedgerows and were considered to be permeable to squirrels. Therefore, it was necessary to digitally add these features to the OSMM. Each field which was comprised of rough grassland or improved land was selected and a field-edge shapefile at a width of 4 m was created and then added to the original OSMM and given a value of 16, the same as scrub, coppice, and garden (see C. D. Stevenson, K. Watts, O. T. Nevin, and A. D. Ramsey, unpubl. data for all land cover resistance values).

### Identifying multiple least-cost paths

**Figure 4:**
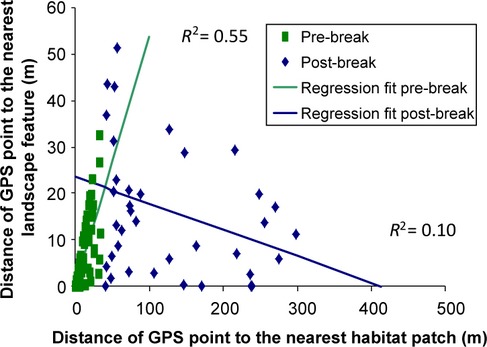
Using regression analysis to show a change in gray squirrel landscape feature at 40 m.

Each woodland patch that contained a GPS location point was selected in ArcGIS. These were used to represent the start and end points of movements. Other woodlands are present in the landscape which were not visited by collared squirrels, but could still facilitate movements. However, in selecting only visited woodlands, it enables a direct comparison between the actual movements and the model outputs. Within ArcGIS, multiple least-cost paths were created based upon pair-wise comparisons. The OSMM data and associated land cover resistance scores that were used in the LCN modeling were also used with the LCP modeling. As the lines are too restrictive to accurately define actual species movement corridors, the LCP lines are then buffered using the buffer tool within ArcGIS with a distance of 40 m either side of the line (Fig. 4). This value was derived from a point-break regression analysis of the distance to the nearest habitat and nearest feature. Each GPS location point was measured to the nearest habitat and feature in the landscape. It indicated that after a distance of 40 m away from habitat, a gray squirrel will orientate towards landscape features to move across the landscape. The buffered LCP were then compared to the actual GPS movement data.

### Identifying least-cost corridors

Least-cost corridors (LCC) were created to enable a further comparison of the LC models. LCC were created by combing the various pair-wise iterations of the ArcGIS LCC tool of the selected woodland patches. As cost surfaces can be difficult to clearly interpret, Singleton et al. (2004) defined the lowest 10% of the cost surface as the LCC. In this study, the LCC is defined by adding 1600 cost units (equivalent of 40 m) to the lowest cost. This figure is based upon an individual being able to move 40 m into the most prominent land cover with a resistance value of 40 (40 × 40 = 1600). The LCC was then compared to the GPS movement data.

### Comparison of LCN, LCP, and LCC

The comparison entailed placing a convex polygon around all the GPS movement points to remove the need to use an arbitrary study area boundary. A test statistic (suggested by a reviewer) was then estimated using the proportion of network, buffered path, or corridor area within the polygon divided by the proportion of points within the network, buffered path, or corridor. In this study, a lower network area with high number of points enables the identification of actual movement paths and is given a low value test statistic which is preferred.

## Results

### GPS telemetry

The GPS devices of two squirrels collared and monitored in the research enclosure before the study remained in the face-up position after 5 and 6 days, respectively. No abnormal behavior was observed and both squirrels were seen to feed and enter traps normally. Examination of the collars did not reveal any significant damage (squirrels have been known to chew through collars of other individuals, but in this case the collars were only fitted for 6 days). In the study, nine gray squirrels above 460 g were captured and released with GPS radio collars fitted. Five squirrels were recaptured (Table 2). The radio collar on squirrel 3 stopped transmitting on the second day after release and therefore the location of this squirrel was not obtained and recapture was not possible. The locations of the remaining squirrels were known, but even though trapping continued until the end of allocated study time they were not recaptured. Eleven noncollared gray squirrels were caught during the recapture period.

**Table 2:** Gray squirrel telemetry data

Squirrel Number	Sex	Weight (g)	Capture date	Recapture date	Days from release
1	Male	535	14/03/2011	22/03/2011	8
2	Female	505	14/03/2011	16/04/2011	33
3	Female	490	15/03/2011	Not recaptured	
4	Male	555	15/03/2011	Not recaptured	
5	Male	525	15/03/2011	21/03/2011	6
6	Male	495	15/03/2011	22/03/2011	7
7	Male	510	15/03/2011	Not recaptured	
8	Male	480	15/03/2011	Not recaptured	
9	Female	675	15/03/2011	07/04/2011	23

### Gray squirrel movements

Clusters of points were highlighted using the GPS data from each collar. A total of 10 interpatch movements were recorded between clusters. The length of the actual movement pathway from the release woodland to the last movement point was significantly longer than the Euclidean distance (*n* = 10, paired *t*-test, *t* = −5.104, df = 9, *P* < 0.001; Table 3).

**Table 3:** GPS squirrel movement data and success of GPS to collect the expected number of location points

Squirrel number	Movement number	Number of locations	Expected number of locations	Euclidean distance (km)	Actual length moved (km)	Time taken (min)	% Location success
1	1	30	42	1.03	1.59	126	71.43
2	1	32	66	0.86	2.11	198	48.48
2	2	29	69	0.24	1.79	207	42.03
5	1	31	42	0.58	2.28	127	73.23
5	2	41	62	2.26	3.47	186	66.13
5	3	15	189	1.19	2.14	568	7.92
6	1	16	27	0.48	0.83	80	60.00
6	2	6	29	1.05	1.18	86	20.93
9	1	18	27	0.48	1.24	80	67.50
9	2	13	13	0.59	0.90	38	100.00

### Use of land cover types and landscape features

**Figure 5:**
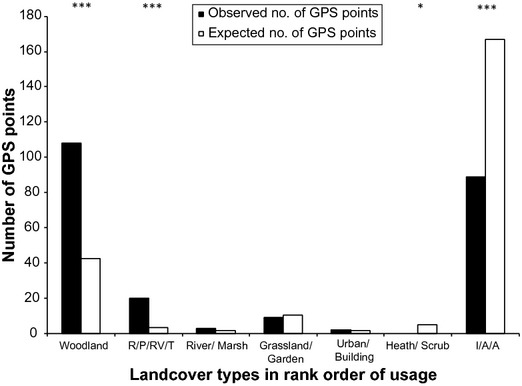
Frequency of GPS points recorded in each land cover type within the study site used in chi-square analysis. I/A/A = Improved/Arable and Amenity, R/P/RV/T = Road/Path/Road Verge/Track, ***P < 0.001 in subdivided testing, **P < 0.001, *P < 0.01.

As expected, a high proportion (47%, *n* = 231) of GPS movement data were recorded in woodland. A large number of GPS movement points (38%, *n* = 231) were recorded in the dominate improved/arable/amenity land cover, however, the amount was much less than expected based on the amount of the landscape available (Fig 5.) and these occurred on the margins of this land cover near either habitat or other landscape features. The remaining 15% of GPS movement points were located in other land cover types, some of which are classed as landscape features (Fig. 5). The number of GPS points within each land cover type was compared with the expected number of GPS points if distributed proportionally to each land cover type using a chi-square goodness–of-fit test. The number of GPS points were not distributed proportionally amongst land cover types (*n* = 231, χ^2^ = 530, df = 6, *P* < 0.001), suggesting a preference for certain land cover types as expected (See Fig. 5).

**Figure 6:**
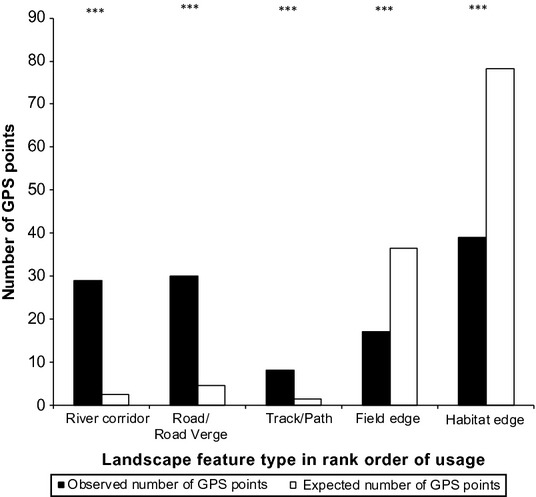
Landscape features that were closest to GPS points used in chi-square analysis. Field edge includes hedgerows, fence rows, and walls, ***P < 0.001 in subdivided testing, **P < 0.001, *P < 0.01.

By measuring the distance from each point that was within the landscape matrix to the nearest habitat patch and comparing these measurements to random points generated in ArcGIS, significantly more GPS points were located nearer a habitat patch then expected by chance (*n* = 124, Mann–Whitney, *U* = 2954, *P* < 0.001). Landscape features within the landscape matrix included: field edge, habitat edge, path, river/stream, road, road verge, and track (Fig. 6). GPS points were also significantly closer to landscape features than expected by chance (*n* = 124, Mann–Whitney, *U* = 3759.5, *P* < 0.001). The use of landscape features was not distributed proportionally among feature types (*n* = 124, χ^2^ = 481.1, df = 4, *P* < 0.001), suggesting a preference for certain landscape features (See Fig 6).

### Comparison of least-cost network analysis, buffered least-cost paths, and least-cost corridor using GPS data

**Figure 7:**
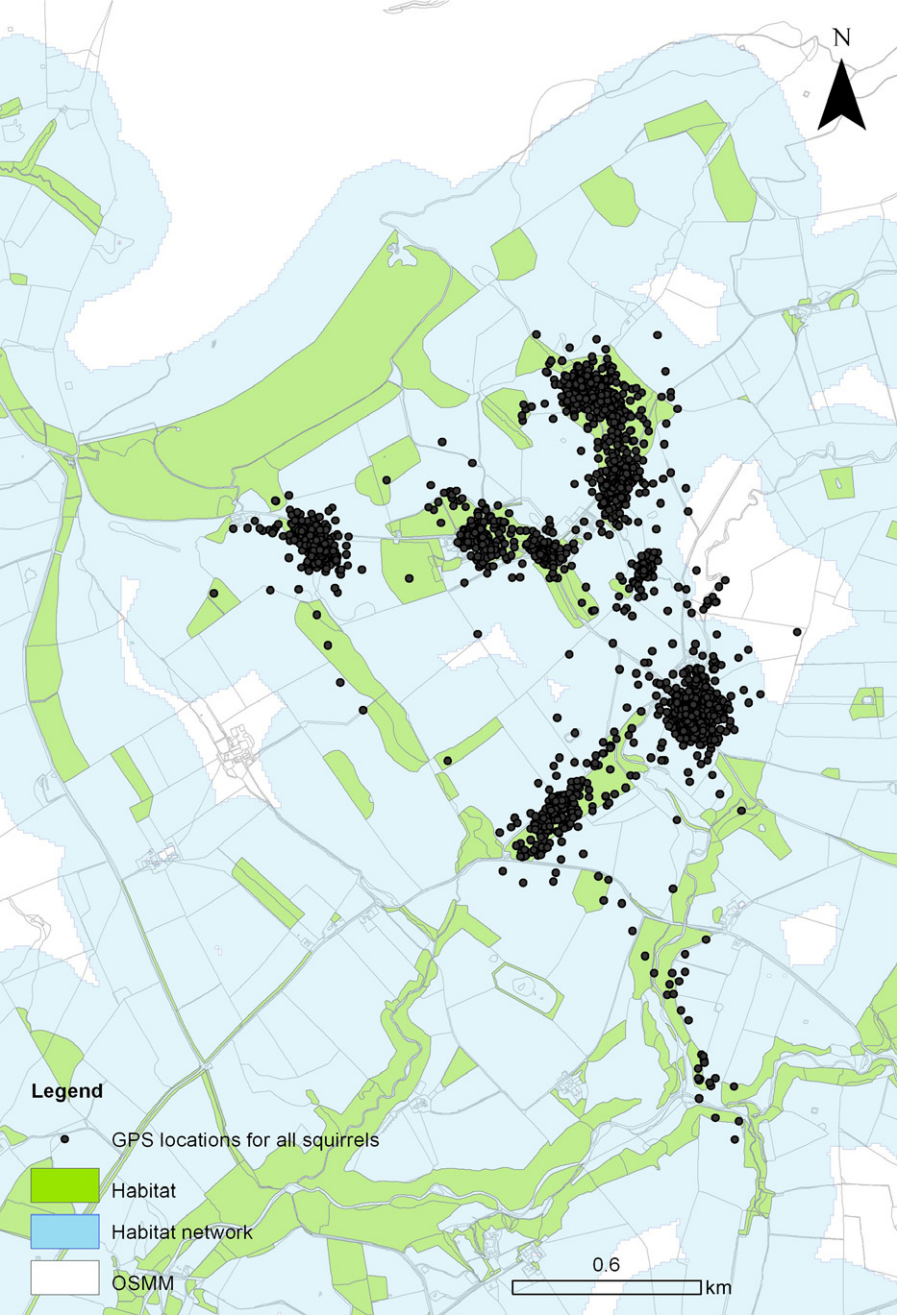
Study site with least-cost networks and GPS data.

The LCN identified a potential large least-cost network within the study which represents areas of the landscape matrix that a gray squirrel is able to move through. The habitat network indicates that the majority of habitat patches within this landscape are functionally connected for the gray squirrel. All the GPS location points recorded on the GPS collars for each of the five recaptured gray squirrels were overlaid on the habitat network (Fig. 7). Using the test statistic of the proportion of least-cost area within the convex polygon divided by the number of points with the network gave a value of 1.01 (97/96 = 1.01).

**Figure 8:**
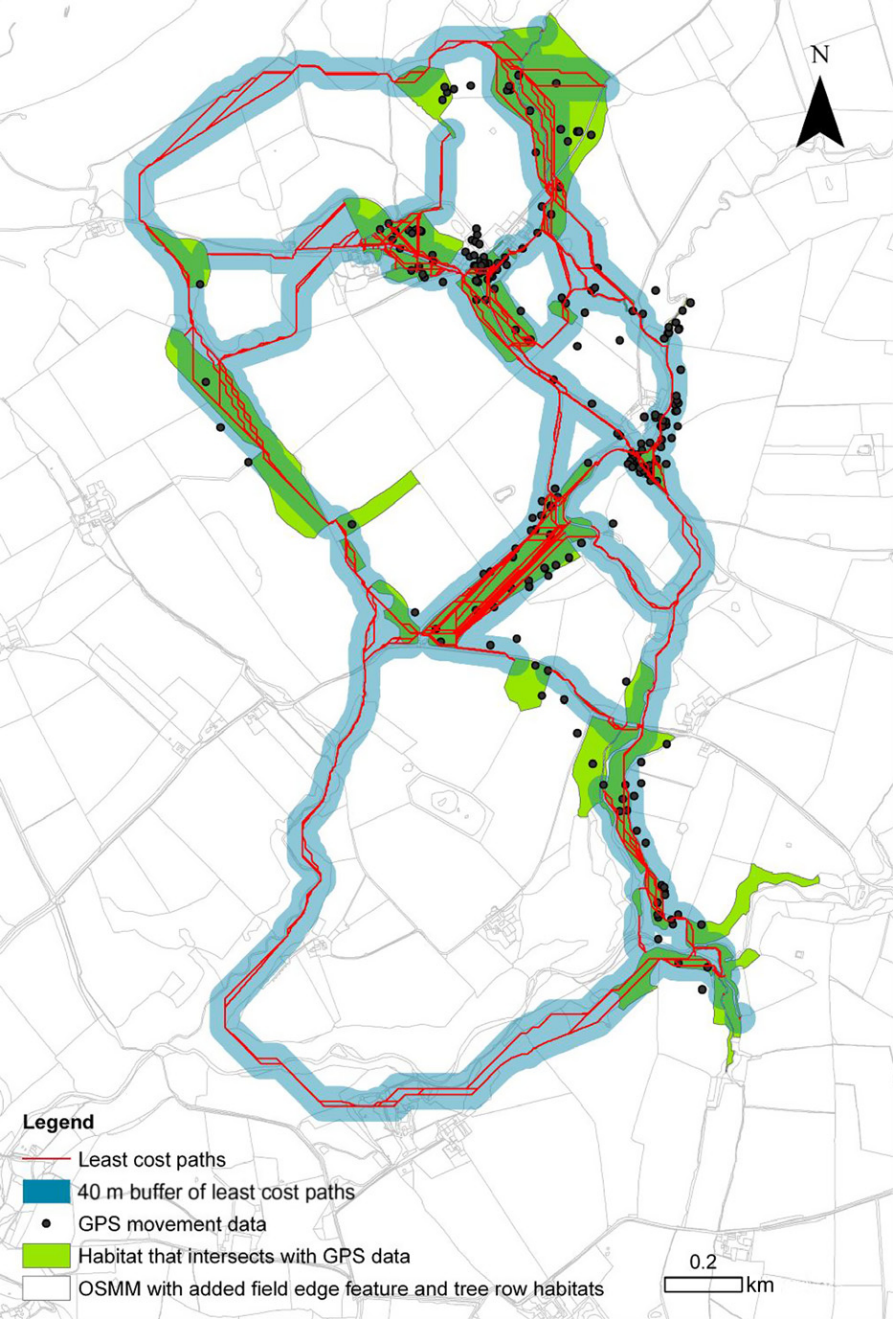
Buffered least-cost paths created from OSMM with additional field edge feature and tree row habitat.

**Figure 9:**
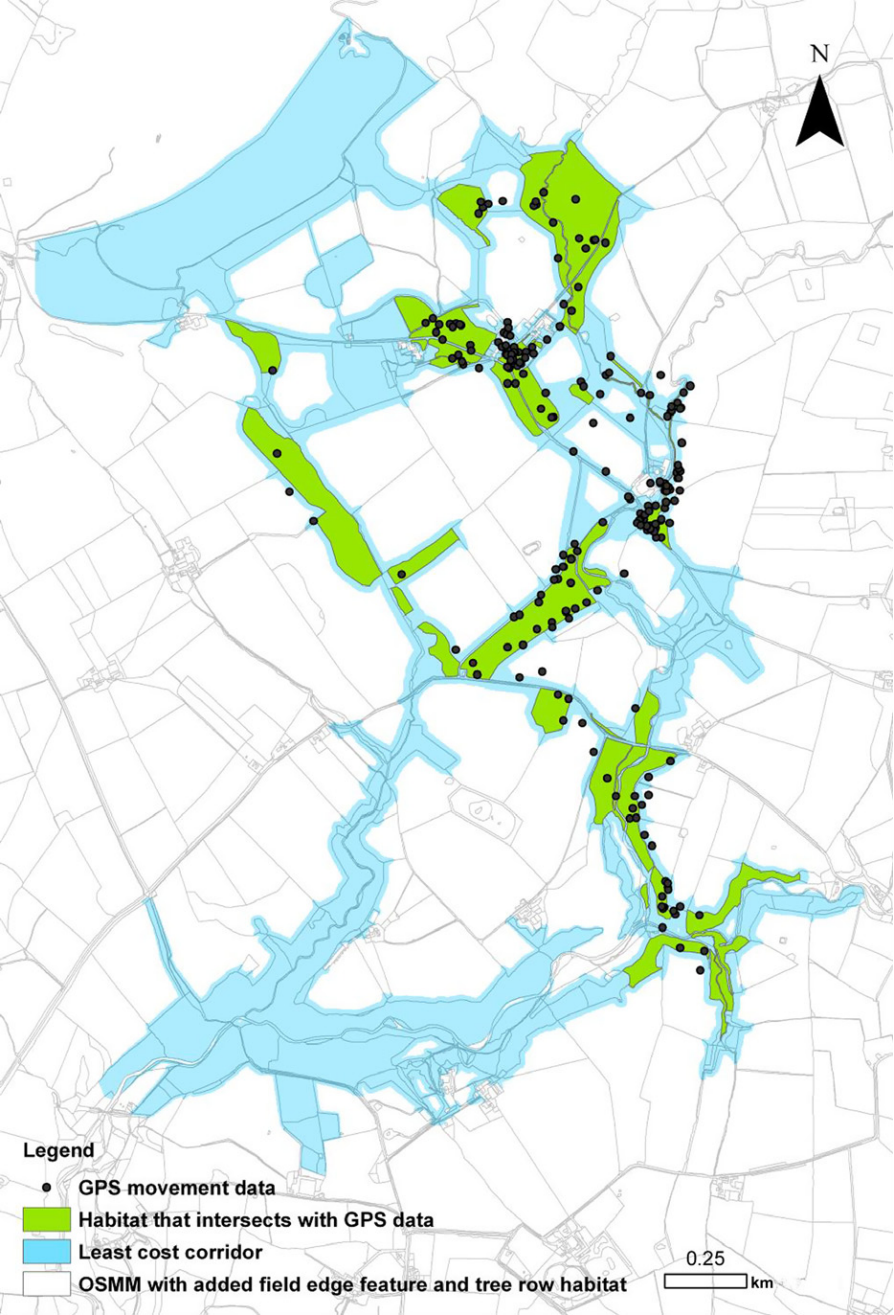
Least-cost corridor identified from OSMM with additional field edge feature and tree row habitats.

The buffered LCP which were based upon OSMM land cover data and with the addition of field edge and woodland patches included 81% (*n* = 231) of the GPS movement data points that were within the landscape matrix and a test statistic of 0.51 (42/81 = 0.51; Fig. 8). The least-cost corridor included 95% of the GPS movement points and a test statistic of 0.51 (49/95 = 0.51; Fig. 9). This value is the same as for LCP and both are much lower than LCN value.

## Discussion

This study has combined LCN, LCP, and LCC modeling techniques to predict gray squirrel movements within the landscape. In addition, GPS movement data were recorded and used to assess the least-cost model predictions. It also contributed to the existing knowledge of gray squirrel landscape use. Previously, recording of a dispersal path using radio telemetry has been used to successfully validate a least-cost model (Driezen et al. 2007). The same study suggested that further validation of least-cost models using different species and landscapes is needed. Previously, GPS telemetry was limited to large animals (Wauters et al. 2007; Haughland et al. 2008; See Tomkiewicz et al. 2010 for a review). However, this is the first study we know which has used GPS collars on in situ gray squirrel individuals to obtain detailed movement data within the landscape matrix.

By using a combination of radio telemetry and GPS, locations were taken for each individual and five of the nine collared gray squirrels were successfully recaptured. Due to time scales, recapture could only take place for a certain time, but gray squirrel control occurs on the study site continuously so it is anticipated that the remaining collared individuals will be recaptured. The data points collected for each squirrel were pooled and used within a chi-square analysis. Although it is suggested that the animal should be used as the test unit and that using each location point in a chi-square analysis causes pseudo replication (Aebischer et al. 1993; Kenward 2001), due to the lower anticipated recovery of individuals, and therefore small samples size using the animal as the test unit would have prejudiced the analysis.

On occasion the GPS device had difficulty in locating enough satellites for signal transfer within the dense canopy, causing a decrease in observation rate (See Rempel et al. 1995). However, the GPS data obtained have enabled assessment of gray squirrel movements within the landscape matrix and 10 interpatch movements have been highlighted. The gray squirrels within this study were translocated <1 km away from the capture site before release. Although the movements recorded with the GPS were not natal dispersal movements, the physiological and behavioral aspects of moving through different land cover types are likely to be similar. Nine out of the 10 movements were directed toward the site of capture. Only one moved in the opposite direction, but changed direction on the same day returning back to where it has started. These movements may possibly suggest a homing instinct of the squirrel back to the capture woodland/home range and show signs of landscape knowledge by the individuals.

Although the types of landscape features and land cover types used were highlighted in the results, it does not show which ones are universally preferred, just the most used within this particular landscape. The availability of land cover types and landscape features is landscape specific and use will depend upon what is available. Land cover type and feature use whilst moving between habitat patches, were ranked based upon the number of points in each compared to the availability of each. Habitat edge is ranked last because during a movement in fragmented landscapes, individuals have to move away from habitat into the landscape matrix. River corridor is ranked first followed by road/road verge then track/path. It has previously been suggested that gray squirrels use landscape features while dispersing (Middleton 1930; Taylor et al. 1971; Fitzgibbon 1993; Bryce et al. 2005). Field edge is ranked second to last most likely because these will be used if other features are not available. By recording the distance to the nearest landscape feature and nearest habitat, the GPS points within this study were found to be significantly closer to habitat patches and landscape features.

The further the individual moves into the landscape matrix and away from habitat, the more susceptible it is to predation and increased energetic costs (Bakker and Vuren 2004). Individuals would be able to perceive a woodland patch if they were within 300 m (Zollner 2000), however, individuals are seen to use landscape features most probably to reduce their risk of predation. This avoidance of open areas behavior has been seen in previous studies (Nixon et al. 1980; Bakker and Vuren 2004) and may have been a consequence of the perceptual range of the individual to detect habitat and predation risk. As the individuals move further from woodland and cannot detect woodland patches in the matrix landscape, features will be used as guidance (Pe'er and Kramer-Schadt 2008). This study reiterates the importance of landscape feature use in gray squirrel movements and shows that features are used, although in doing so the distance traveled is longer.

When the Euclidean distance was compared with the actual movement distance, individuals were seen not to take the straightest distance between two woodland patches. Movements were significantly longer and included the use of landscape features. Chardon et al. (2003) and Verbeylen et al. (2003) both suggested that presence and absence data were better explained by a least-cost model than the Euclidean distance. Coulon et al. (2004) and Driezen et al. (2007) showed that genetic distance and radio telemetry data, respectively, also validated least-cost paths. Within this study significantly more GPS movement points were within the buffered paths and corridors than expected by chance. The results indicate that the least-cost modeling approach not only predicts movements better than the Euclidean distance but it also is able to successfully predict gray squirrel dispersal within the landscape matrix.

The GPS data were used to validate the LCN, buffered LCP, and LCC created with the OSMM. The scale and quality of the base maps used within least-cost modeling has an impact on the model outputs (Adriaensen et al. 2003; Sawyer et al. 2011). It is essential that the accuracy of the map is considered and all landscape elements which are important to the dispersal of the study species are included within the base map at an appropriate scale (Villalba et al. 1998; Verbeylen et al. 2003). If they are not included, extra digitization is required (Schadt et al. 2002; Adriaensen et al. 2003; Verbeylen et al. 2003). Hedgerows, walls, and fences, which are classed as field edges in this study, are seen to be important to gray squirrel dispersal (Middleton 1930; Taylor et al. 1971; Fitzgibbon 1993; Bryce et al. 2005), and therefore it was important to add these additional features and missing habitat to the base map.

Each of the least-cost modeling techniques used within this study provide information on the functional connectivity of gray squirrel habitat within the landscape. By using a combination of LCN, buffered LCP, and LCC modeling, an apparent progression can be seen. On the larger spatial scale, the networks identify areas of the landscape in which a species is able to disperse. This can cover substantial areas and includes all areas not just the most probable routes. To predict the most probable routes, a gray squirrel would move, the next step is to use multiple buffered LCP, LCC, or both, to gain fine-scale movements within networks. Buffered LCP's are relatively quick to produce and were assessed using GPS movement data. Although the test statistic produced the same values for LCP and LCC, LCC does accommodate variation in widths which is biologically more realistic.

Based upon previous literature and expert knowledge, Gurnell et al. (2006) used a spatial explicit population model to highlight gray squirrel incursion routes into Kielder forest, a red squirrel reserve. The model used by Gurnell et al. (2006) suggested that dispersal into the forest occurred through the use of narrow river valleys with hedgerows and woodland patches (Gurnell et al. 2006). This study has shown that least-cost modeling is also capable of predicting gray squirrel movements in the landscape. The next step will be to use least-cost modeling to identify the most probable gray squirrel movement routes in areas where red squirrel conservation occurs. This will enable gray squirrel control to be targeted in specific areas aiding their management. By using a combination of LCN, buffered LCP, and LCC modeling to assess the functional connectivity of habitat patches for the gray squirrel, potential dispersal routes have been identified.

This is the first study to use GPS telemetry on gray squirrel. Although it is acknowledged that a small number of individuals were collared, it has shown that this technique is successful in gaining information on movement to enable least-cost model validation. The techniques used within this study can be applied to different species and landscapes in addition to other conservation and management strategies. Potentially, these techniques can be used to aid red squirrel conservation and gray squirrel management by highlighting potential movement routes through the landscape.
